# MHD darcy-forchheimer nanofluid flow and entropy optimization in an odd-shaped enclosure filled with a (MWCNT-Fe_3_O_4_/water) using galerkin finite element analysis

**DOI:** 10.1038/s41598-021-02047-y

**Published:** 2021-11-22

**Authors:** Wael Al-Kouz, Abderrahmane Aissa, Aimad Koulali, Wasim Jamshed, Hazim Moria, Kottakkaran Sooppy Nisar, Abed Mourad, Abdel-Haleem Abdel-Aty, M. Motawi Khashan, I. S. Yahia

**Affiliations:** 1 Mechanical and Maintenance Engineering Department, School of Applied Technical Sciences, German Jordanian University, Amman, 11180 Jordan; 2 Laboratoire de Physique Quantique de la Matière et Modélisation Mathématique (LPQ3M), Université Mustapha Stambouli de Mascara, Mascara, Algeria; 3 Department of Mathematics, Capital University of Science and Technology (CUST), Islamabad, 44000 Pakistan; 4 Department of Mechanical Engineering Technology, Yanbu Industrial College, Yanbu Al-Sinaiyah City, 41912 Kingdom of Saudi Arabia; 5 Department of Mathematics, College of Arts and Sciences, Prince Sattam Bin Abdulaziz University, Wadi Aldawaser, 11991 Saudi Arabia; 6 Department of Physics, College of Sciences, University of Bisha, P.O. Box 344, Bisha, 61922 Saudi Arabia; 7 Physics Department, Faculty of Science, Al-Azhar University, Assiut, 71524 Egypt; 8 Department of Basic Sciences, Common First Year, King Saud University, Riyadh, 11451 Saudi Arabia; 9 Advanced Functional Materials & Optoelectronic Laboratory (AFMOL), Department of Physics, Faculty of Science, King Khalid University, P.O. Box 9004, Abha, Saudi Arabia; 10 Research Center for Advanced Materials Science (RCAMS), King Khalid University, P.O. Box 9004, Abha, 61413 Saudi Arabia; 11 Nanoscience Laboratory for Environmental and Biomedical Applications (NLEBA), Semiconductor Lab., Department of Physics, Faculty of Education, Ain Shams University, Roxy, 11757 Cairo Egypt

**Keywords:** Mathematics and computing, Physics

## Abstract

MHD nanoliquid convective flow in an odd-shaped cavity filled with a multi-walled carbon nanotube-iron (II, III) oxide (MWCNT-Fe_3_O_4_) hybrid nanofluid is reported. The side walls are adiabatic, and the internal and external borders of the cavity are isothermally kept at high and low temperatures of Th and Tc, respectively. The governing equations obtained with the Boussinesq approximation are solved using Galerkin Finite Element Method (GFEM). Impact of Darcy number (Da), Hartmann number (Ha), Rayleigh number (Ra), solid volume fraction (ϕ), and Heated-wall length effect are presented. Outputs are illustrated in forms of streamlines, isotherms, and Nusselt number. The impact of multiple parameters namely Rayleigh number, Darcy number, on entropy generation rate was analyzed and discussed in post-processing under laminar and turbulent flow regimes.

## Introduction

At the end of the twentieth century, a new class of fluid emerged owing to the work done by a team of researchers led by professor Choi^1^, who named it nanofluid. This new engineered fluid exhibited exceptional heat transfer characteristics and offered the great benefit of enhancing any thermal system performance without any modifications to its components. Nowadays we can find nanofluids in a plethora of energy, bioengineering, and industrial applications thanks to the work done by many researchers on nanofluid to expand their utility for example in heat exchangers^2^, solar collectors^3^, material engineering^4^, energy storage systems^5^, engine oil^6^, Bio-technology^7^ and Water Cleaning Process^8^.

Over the past two decades, several studies have been published on nanofluids and their flow and heat transfer behaviors. Rashmi^9^ found gains of 6.3% and 18.45% in the second law of efficiency and heat transfer rate, respectively when he tested a ternary hybrid nanoliquid as a radiator coolant. He also noted that the performance of this hybrid nanofluid was dependent on the volume concentration and the shape of the solid particles. Choi et al.^10^ discussed enhancing the performance of a radiator used to cool a 100 kW high power system by employing an EG/water-based Al_2_O_3_ nanoliquid they found that the nanofluid enhanced the heat transfer rate inside the radiator by 6.9%. Moreover, they demonstrated the nanofluid they used, could be mass-produced and that its long-term suspension stability was well maintained during the study period. Ahmadi et al.^11^ studied the influence of employing nanoliquid in a shell and tube heat exchanger cooling an EGR system of a diesel engine. Desouky et al*.*^12^ examined numerically the MHD thermal behavior inside a T-shaped enclosure filled with nanofluid and under the influence of Lorentz force and the motion of the upper and lower parallel walls Merino et al.^13^ provided an insight into how the method of nanofluid preparation affects its thermal performance and stability. Fadodun et al*.*^14^ examined the heat transport rate of an Al_2_O_3_water-based nanofluid as it circulated inside a converging pipe. Suspending nanoparticles in a base fluid has been proven to be an effective technique in augmenting the thermal performance in various thermal applications^15–19^.

The analysis of nanofluid flow through porous media has been receiving tremendous attentiveness from many researchers and the most common model used in this type of study is the Darcy model developed by Henry Darcy in 1856^20^. Taking into consideration the 1 and 2 laws of thermodynamics, Shahsavar et al.^21^ evaluate the hydrothermal performance of a heat sink filled with metal foam and saturated by an eco-friendly water-silver nanofluid. They stated that although the presence of porous medium diminished the entropy production rate, it improved the overall thermal performance of the heat sink. Alihosseini et al.^22^ observed that the thermal performance of nanofluid when flowing through a cylinder fully saturated with a porous medium was better when compared to its performance in an empty cylinder. Th. Benos et al*.*^23^ researched the hydrothermal characteristics of nanofluid inside a rectangular porous enclosure subjected to an external uniform magnetic field and an internal heating source. Baïri et al.^24^ discussed the enhancement of the performance of a spherical thermal management system for a spherical electronic device, using permeable media filled with a nanofluid. Liu et al.^25^ simulated the hydrothermal behavior of water-based Cu nanoliquid saturating an annulus filled with porous media. Tahmasbi et al*.*^26^ analyzed the Mixed convection of nanofluid inside a square enclosure filled with optimized permeable media and equipped with two rotating cylinders. Using the non-equilibrium technique, Shafee et al*.*^27^ scrutinized the impact of a magnetic field on the heat transfer of a nanoliquid within a cavity filled with porous media. They found that the Lorentz forces suppressed convective flow. Salari et al*.*^28^ studied experimentally the influence of using nanofluids to enhance a heat exchanger partially filled with a permeable medium. The outcomes show that using both the nanofluid and the partial porous medium contributed to the improvement of the thermal performance rate in the heat exchanger. Aminian et al*.*^29^ directed a numerical investigation on MHD forced convective heat transport of a nanofluid flowing through a cylinder saturated with a porous medium. In addition, they defined a performance evaluation criterion (PEC) to compare the thermal and hydrodynamic performance of the different configurations of the investigated system. According to their results, the Hartmann and Darcy number have an undeniable positive impact on the enhancement of the PEC and heat transfer rate.

Furthermore, several scientists reported on the entropy production of nanofluid in various cavities and under various conditions for example inside a square cavity and under a magnetic field Kefayati et al*.*^30^, an enclosure with wavy side walls filled with a ferrofluid Afsana et al*.*^31^. Bahiraei^32^ studied the heat transportation performance of a hybrid eco-friendly nanoliquid flowing inside tubes equipped with rotary twisted tape. They found that employing the twisted tape at relatively high rotation speeds, (around 900 rpm) greatly diminished the total entropy production and total exergy destruction of the hybrid nanolquid. In another study by Bahiraei et al*.*^33^ inspected the entropy production for the flow of a hybrid nanoliquid through a microchannel heat sink outfitted with secondary channels and ribs. Ma et al*.*^34^ numerically studied the first and second law performance of a branching microchannels heatsink employing ecofriendly Ag-water based nanofluid, using a two-phase mixture model. They also discussed the impact of various fin arrangements. In addition to the previously mentioned literature review, it should be mentioned that other authors have given an extensive reviews and they investigated both forced and natural convection where the nanofluid was utilized for conventional and rarefied flows under different physical and geometrical effects for different applications. These include but not limited to MHD, porous media, fins, internal heat generation and many others^35–49^.

The present work investigates MHD natural convection response within an Odd-Shaped enclosure Filled with a (MWCNT-Fe_3_O_4_/ H_2_O). Several parameters are studied such as Ra $\left({10}^{3}\le \mathrm{Ra}\le {10}^{6}\right).$ Ha $(0\le \mathrm{Ha}\le 100)$, and Da $\left({10}^{-5}\le \mathrm{Da}\le 0.15\right)$. 2D numerical simulation is performed on this geometry using the finite element method and directed to identify the optimum conditions for obtaining the best heat transfer possible through this configuration.

### Physical model

**Figure 1:**
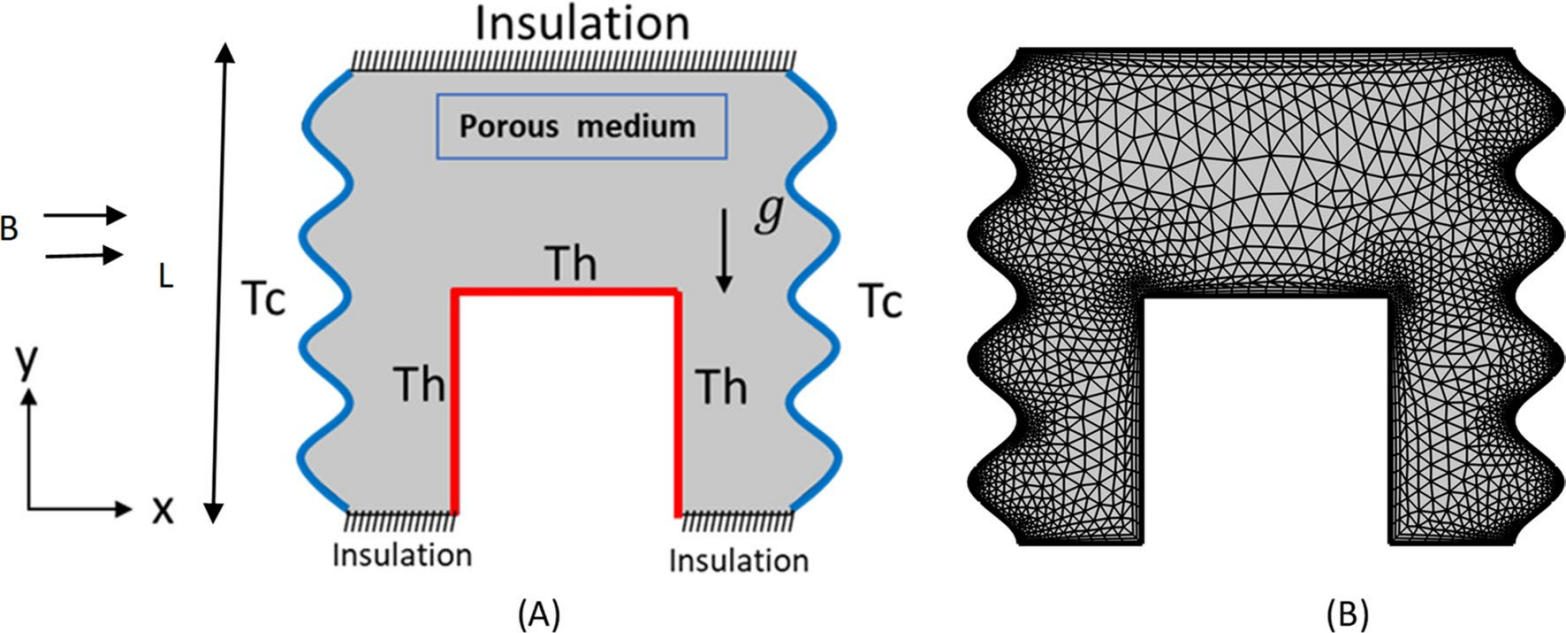
Schematic illustration of corrugated enclosure and sample mesh.

As mentioned in the current text, the problem description is based on magnetic force's influence on the nanoparticle treatment process within the medium, taking into account the impact of permeability. More specifically, the objective is to see how to change the flow condition parameters, such as the radiation parameter, Ra and Ha numbers, the nanoparticle content, and the number of corrugations, with a view to controlling heat transfer and entropy generation in the domain. Here, the physical model (see Fig. 1) is a trapezoidal cavity cooled by the sides (Tc) and heated by a corrugated bottom (Th). The upper wall of the cavity is considered adiabatic. Table 1 exhibits characteristics of hybrid nanofluid.

**Table 1:** Thermo physical properties of water and nanoparticles^50^.

	$\rho$(kg/m^3^)	C_p_ (J/kg k)	k (W/m k)	σ (S/ m)	β (K ^-1^)
Pure water	997.1	4179	0.613	5.5 × 10^–6^	21 × 10^–5^
Fe_3_O_4_	5810	670	6	2.5 × 10^–4^	1.3 × 10^–5^
MWCNT	2100	711	3000	1.9 × 10^–4^	4.2 × 10

The wavy walls equations obey.

1 $$x = - A\sin (\frac{N\pi x}{H})$$

The thermophysical properties of water and Multi-Walled Carbon Nanotube-Iron oxide (MWCNT-Fe_3_O_4_) are defined in Table 1.

## Mathematical formulation

The porous cavity is filled with Fe_3_O_4_/MWCNT-water hybrid nanoliquid. The Forchheimer-Brinkman-extended^51^ Darcy model is adopted together with the Boussinesq approximation:

2 $$\frac{\partial u}{{\partial x}} + \frac{\partial v}{{\partial y}} = 0$$

3 $$\frac{{\rho_{hnf} }}{{\varepsilon^{2} }}\left( {u\frac{\partial u}{{\partial x}} + v\frac{\partial u}{{\partial y}}} \right) = - \frac{\partial P}{{\partial x}} + \frac{{\mu_{hnf} }}{\varepsilon }\left( {\frac{{\partial^{2} u}}{{\partial x^{2} }} + \frac{{\partial^{2} u}}{{\partial y^{2} }}} \right) - \left( {\frac{{\mu_{hnf} }}{K}u - \frac{1.75}{{\sqrt {150} \left( \varepsilon \right)^{\frac{3}{2}} }}\frac{{\mu_{hnf} u\left| u \right|}}{\sqrt K }} \right)$$

4 $$\frac{{\rho_{hnf} }}{{\varepsilon^{2} }}\left( {u\frac{\partial v}{{\partial x}} + v\frac{\partial v}{{\partial y}}} \right) = - \frac{\partial p}{{\partial y}} + \frac{{\mu_{hnf} }}{\varepsilon }\left( {\frac{{\partial^{2} v}}{{\partial x^{2} }} + \frac{{\partial^{2} v}}{{\partial y^{2} }}} \right) - \left( {\frac{{\mu_{hnf} }}{K}u - \frac{1.75}{{\sqrt {150} \left( \varepsilon \right)^{\frac{3}{2}} }}\frac{{\mu_{nf} u\left| u \right|}}{\sqrt K }} \right) + \left( {\rho \beta } \right)_{hnf} g(T_{h} - T_{c} ) - \sigma_{hnf} B_{0} v^{2}$$

5 $$u_{hnf} \frac{{\partial T_{nhf} }}{\partial x} + v_{hnf} \frac{{\partial T_{hnf} }}{\partial y} = \alpha_{hnf} \left( {\frac{{\partial^{2} T_{hnf} }}{{\partial x^{2} }} + \frac{{\partial^{2} T_{hnf} }}{{\partial y^{2} }}} \right)$$

The volume fraction of nanoparticle types used are formulated as follows:

6 $$\varphi = \varphi ({\text{MWCNT}}) \, + \varphi \left( {{\text{Fe}}_{{3}} {\text{O}}_{{4}} } \right)$$

The following nanofluid thermophysical properties are utilized^51–57^:

7 $$\left\{ \begin{gathered} \rho_{hnf} = (1 - \phi )\rho_{f} + \phi \rho_{p} \hfill \\ (\rho \beta )_{hnf} = (1 - \phi )(\rho \beta )_{f} + \phi (\rho \beta )_{p} \hfill \\ \end{gathered} \right.$$

8 $$\left\{ \begin{gathered} (\rho C_{p} )_{hnf} = (1 - \phi )(\rho C_{p} )_{f} + \phi (\rho C_{p} )_{p} \hfill \\ \alpha_{hnf} = \frac{{k_{hnf} }}{{(\rho C_{p} )_{hnf} }} \hfill \\ \end{gathered} \right.$$

9 $$\frac{{k_{hnf} }}{{k_{f} }} = \frac{{k_{np} + \left( {n - 1} \right)k_{f} - \left( {n - 1} \right)\left( {k_{f} - k_{np} } \right)\varphi }}{{k_{np} + \left( {n - 1} \right)k_{f} + \left( {k_{f} - k_{np} } \right)\varphi }}$$

10 $$\mu_{hnf} = \frac{{\mu_{{_{bf} }} }}{{\left( {1 - \varphi } \right)^{2.5} }}$$

The following dimensionless variables are utilized:

11 $$X = \frac{x}{L},Y = \frac{y}{L}$$

12 $$U = \frac{uL}{{\alpha_{f} }},V = \frac{vL}{{\alpha_{f} }}$$

13 $$\theta_{nf} = \frac{{T_{nf} - T_{c} }}{{T_{h} - T_{c} }}, \quad \theta_{s} = \frac{{T_{s} - T_{c} }}{{T_{h} - T_{c} }}, \quad \Pr = \frac{{\nu_{f} }}{{\alpha_{f} }}$$

$$Ra = \frac{{g\beta_{f} (T_{h} - T_{c} )L^{3} }}{{\nu_{f} \alpha_{f} }},{\text{ P = }}\frac{{pL^{2} }}{{\rho_{f} \alpha_{f}^{2} }},{\text{ k}}_{eff} = \varepsilon k_{nf} + (1 - \varepsilon )k_{m} ,{\text{ C}}_{F} = \frac{1.75}{{\sqrt {150} }}$$

$$Da = \frac{\lambda }{{L^{2} }}, \quad \Pr = \frac{{v_{fl} }}{{\alpha_{fl} }}$$

14 $$Ha = LB_{{}} \sqrt {\frac{{\sigma_{nf} }}{{\mu_{nf} }}}.$$

As a result, we have

15 $$\frac{\partial U}{{\partial X}} + \frac{\partial V}{{\partial Y}} = 0$$

16 $$\begin{gathered} \frac{1}{{\varepsilon^{2} }}\left( {U\frac{\partial U}{{\partial X}} + V\frac{\partial U}{{\partial Y}}} \right) = - \frac{\partial P}{{\partial X}} + \frac{{\rho_{f} }}{{\rho_{hnf} }}\frac{{\mu_{hnf} }}{{\mu_{f} }}\frac{\Pr }{\varepsilon }\left( {\frac{{\partial^{2} U}}{{\partial X^{2} }} + \frac{{\partial^{2} U}}{{\partial Y^{2} }}} \right) \hfill \\ \quad - \frac{{\rho_{f} }}{{\rho_{hnf} }}\frac{{\mu_{hnf} }}{{\mu_{f} }}\frac{\Pr }{{Da}}U - \frac{{C_{F} \sqrt {U^{2} + V^{2} } }}{{\sqrt {Da} }}\frac{U}{{\varepsilon^{3/2} }} \hfill \\ \end{gathered}$$

17 $$\begin{gathered} \left( {U\frac{\partial V}{{\partial X}} + V\frac{\partial V}{{\partial Y}}} \right) = - \frac{\partial P}{{\partial X}} + \frac{{\rho_{f} }}{{\rho_{hnf} }}\frac{{\mu_{hnf} }}{{\mu_{f} }}\frac{\Pr }{\varepsilon }\left( {\frac{{\partial^{2} V}}{{\partial X^{2} }} + \frac{{\partial^{2} V}}{{\partial Y^{2} }}} \right) - \frac{{\rho_{f} }}{{\rho_{nf} }}\frac{{\mu_{nf} }}{{\mu_{f} }}\frac{\Pr }{{Da}}V - \frac{{C_{F} \sqrt {U^{2} + V^{2} } }}{{\sqrt {Da} }}\frac{V}{{\varepsilon^{3/2} }} \hfill \\ \quad + \frac{{(\rho \beta )_{hnf} }}{{\rho_{hnf} \beta_{f} }}Ra\Pr \theta - Ha^{2} \Pr \frac{{\sigma_{hnf} }}{{\sigma_{f} }}\frac{{\rho_{f} }}{{\rho_{hnf} }}V \hfill \\ \end{gathered}$$

18 $$U\frac{\partial \theta }{{\partial X}} + V\frac{\partial \theta }{{\partial Y}} = \alpha_{hnf} \left( {\frac{{\partial^{2} \theta }}{{\partial X^{2} }} + \frac{{\partial^{2} \theta }}{{\partial Y^{2} }}} \right)$$

### Boundary conditions

The boundary conditions now become.

#### For top wall

19 $$U = V = 0, \quad \frac{{\partial \theta_{nf} }}{\partial Y} = 0$$

#### Heated part of inner wall

$${\text{U = V = 0, }} \quad \theta_{nf} = 1, \,$$

#### For outer wall

$${\text{U = V = 0, }} \quad \theta_{nf} = 0, \,$$

The average numbers are defined as

20 $$Nu_{avg} = \frac{{k_{nf} }}{{k_{f} }}\frac{\partial \theta }{{\partial n}}$$

### Entropy production analysis

The entropy production relation is given by^47,48^:

21 $${S}_{T}=\frac{{k}_{nf}}{{T}_{0}^{2}}\left[{\left(\frac{\partial T}{\partial x}\right)}^{2}+{\left(\frac{\partial T}{\partial y}\right)}^{2}\right]+\frac{{\mu }_{nf}}{{T}_{0}}\left[2{\left(\frac{\partial u}{\partial x}\right)}^{2}+2{\left(\frac{\partial v}{\partial y}\right)}^{2}+{\left(\frac{\partial u}{\partial x}+\frac{\partial v}{\partial x}\right)}^{2}\right]+\frac{{\sigma }_{nf}{B}_{0}^{2}}{{T}_{0}}\left({u}^{2}+{v}^{2}\right).$$

In dimensionless form can be expressed as:

22 $${S}_{T}=\frac{{k}_{nf}}{{k}_{f}}\left[{\left(\frac{\partial \theta }{\partial X}\right)}^{2}+{\left(\frac{\partial \theta }{\partial Y}\right)}^{2}\right]+\frac{{\mu }_{nf}}{{\mu }_{f}}\upchi \left\{2\left[{\left(\frac{\partial U}{\partial X}\right)}^{2}+2{\left(\frac{\partial V}{\partial Y}\right)}^{2}\right]+{\left(\frac{\partial U}{\partial Y}+\frac{\partial V}{\partial X}\right)}^{2}+\upchi {\mathrm{Ha}}^{2}\frac{{\sigma }_{nf}}{{\sigma }_{f}}({U}^{2}+{V}^{2})\right\}.$$

where,

23 $$\upchi =\frac{{\mu }_{f}{T}_{0}}{{k}_{f}}{\left(\frac{{u}_{w}}{{T}_{h}-{T}_{c}}\right)}^{2},$$

is the irreversibility distribution ratio and The terms of Eq. (27) can be separated into the following form:

24 $${S}_{T}={S}_{HT}+{S}_{FF}+{S}_{MF},$$

where ${S}_{HT}$ , S_FF_ and S_MF_ are the entropy production due to heat transfer irreversibility (HTI), fluid friction irreversibility (FFI) and magnetic field (MF) respectively.

25 $${S}_{HT}=\frac{{k}_{nf}}{{k}_{f}}\left[{\left(\frac{\partial \theta }{\partial X}\right)}^{2}+{\left(\frac{\partial \theta }{\partial Y}\right)}^{2}\right]$$

26 $${S}_{FF}=\frac{{\mu }_{nf}}{{\mu }_{f}}\upchi \left\{2\left[{\left(\frac{\partial U}{\partial X}\right)}^{2}+2{\left(\frac{\partial V}{\partial Y}\right)}^{2}\right]+{\left(\frac{\partial U}{\partial Y}+\frac{\partial V}{\partial X}\right)}^{2}\right\}.$$

27 $${S}_{MF}=\upchi {\mathrm{Ha}}^{2}\frac{{\sigma }_{nf}}{{\sigma }_{f}}({U}^{2}+{V}^{2})$$

Bejan number is defined as:

28 $${B}_{e}=\frac{\int {S}_{HT}\mathrm{dXdY}}{\int {S}_{T}\mathrm{dXdY}}=\frac{{S}_{HT}}{{S}_{T}}$$

## Method of solution

### Validation and grid independence

**Figure 2:**
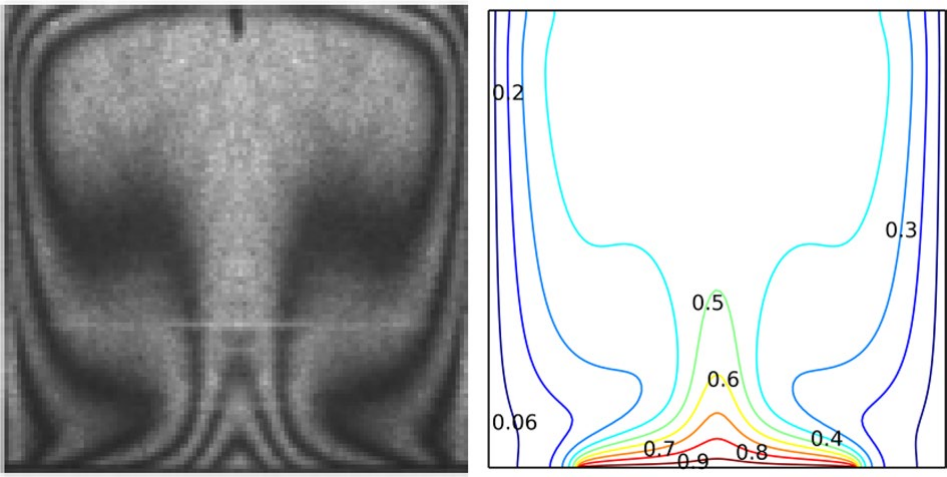
Validation of numerical code with Calcagni et al.^53^ at Ra = 10^5^.

Galerkin weighted residual finite element method was used for the solution of the governing equations along with the boundary conditions. several grids are tested. As indicated in Table 2, The obtained results lead us to consider the extra-fine grid with 40,600 triangular elements to be used in the current study. To assure the accuracy of the numerical method of the adopted code, the isothermal contours are represented and then compared with published results obtained by Calcagni et al.^58^ as showed in Fig. 2.

**Table 2:** Grid sensitivity check (Ha = 0, φ = 0.04, Da = 10^–2^, Ra = 10^5^).

	940	1534	2442	10,920	40,600	43,900
Nu_avg_	8.8197	9.1230	9.3964	10.299	**11.207**	11.200
$\psi_{\max }$	2.4309	2.4309	2.4437	2.4504	**2.451**	2.4520

## Results and discussion

In this part, we discuss in detail the results obtained from the numerical simulations carried out in this work. The results have been given in terms of streamline, isotherms, and Isentropic contours within the odd-shaped cavity in question. Also, we presented the average Nusselt number variation a Bejan number evolution as a function of control parameters at the hot wall. In this work, the effects of a wide range of parameters on flow structure, temperature distribution, and induced entropy generation have been examined. The volume fraction of nanoparticles within the fluid domain was set at $\varphi$=0.04, gravity effects due to temperature differences inside the porous cavity were varied using a wide range of Ra (10^3^ $\le Ra\le$ 10^6^). Also, the magnetic field impact was considered with (0 $\le Ha\le$ 100), besides the Darcy number influence was taken into account using (10^–5^ $\le Da\le$ 10^–2^). The geometrical shape of the cavity was examined by considering different lengths of the heated wall.

### Flow field, temperature distribution, and entropy generation

#### Rayleigh number effect

**Figure 3:**
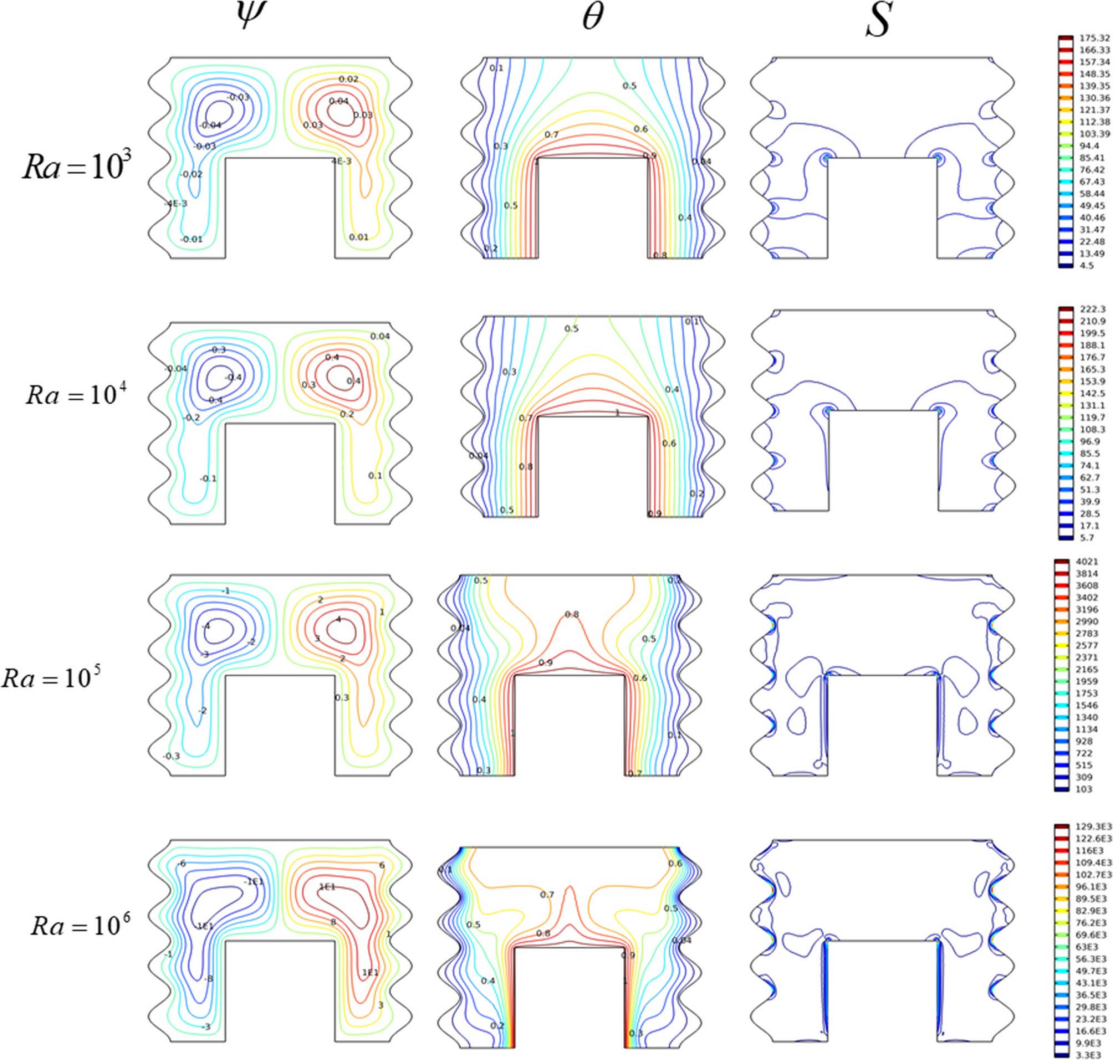
The impact of Ra on (ψ) Streamlines, (θ) Isotherms, and (S) Entropy for Da = 10^–2^, Ha = 0.

Without magnetic force (Ha = 0) and under (Da = 10^–2^), we investigated Rayleigh number effects on streamlines, isotherms, and entropy production. The results of this examination can be seen in Fig. 3. In such structures, the flow force within the cavity occurs by virtue of thermal thrust caused by the temperature gradient (T_h_-T_c_) between the convex wall and the lateral walls of the cavity. The symmetrical boundary conditions imposed on the odd-shaped cavity walls create a symmetrical flow structure with respect to the vertical axis passing through the cavity center (X = 0.5). Indeed, a bi-cellular flow pattern prevails with an upward movement at the center of the cavity. Under slow flow regimes (Ra = 10^3^), convection streams are very weak ($\psi_{\max }$** = **0.04), and uniform temperature distribution occupies the whole domain. In this case thermal conduction governs both flow and temperature distribution structures. Isothermal line values increase gradually from the hot wall to the cold wall. This topology, called thermal stratification, is one of the characteristics of slow flows whose heat transfer occurs mainly by thermal diffusion.

In this case, low entropy generation (4.5 $\le$ **S** $\le$ 175.3) is observed at the cones of the convex hot wall of the cavity, due to the weak condensation of isotherms near this region. Increased Rayleigh numbers are accompanied by higher buoyancy forces Ra = 10^6^. Therefore, a more important fluid circulation is required. The structure remains bi-cellular except that the shape of cells changes and they take an oval shape, indicating an increase in the induced flow. In this case, convection takes over and is accompanied by distortion of the isotherms which appear as thermal plumes. The latter is a sign of more intense convection currents ($\psi_{\max }$ = 12.3), which promotes the convection mechanism over that of conduction. The cold walls and the warm convex wall exhibit greater thermal gradients, creating active regions for local entropy generation. So, we notice that the produced entropy is more important in the case of high Rayleigh numbers with (3.3 × 10^3^ $\le$ S $\le$ 1.29 × 10^5^).

#### Hartman number effect

**Figure 4:**
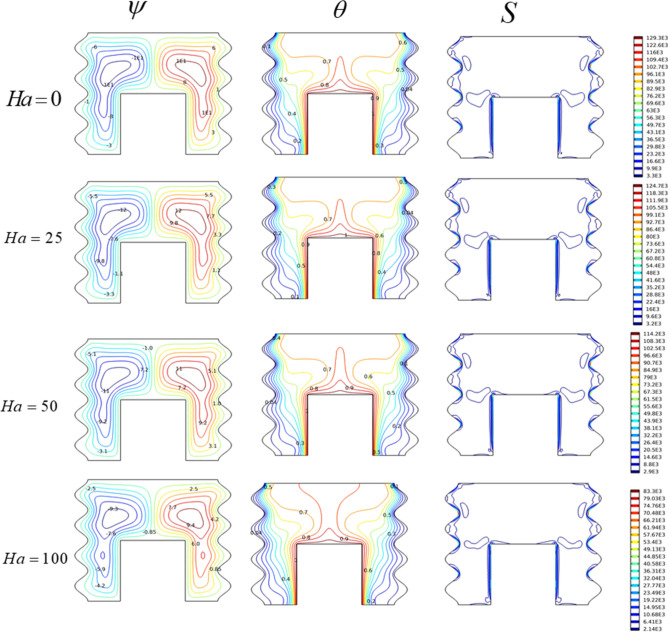
The impact of Ha on (ψ) Streamlines, (θ) Isotherms, and (S) Entropy for Da = 10^–2^, Ra = 10^6^.

The influence of the electromagnetic forces exerted by the magnetic field on the nanofluid under investigation was examined through the Hartman number in a range of (0 $\le$ Ha $\le$ 100), with (Ra = 10^6^, Da = 10^–2^, and AR = 0.5). Figure 4 depicts the current lines, isotherms, and entropy production as a function of the Hartman number. Without a magnetic field (Ha = 0), the flow occurs as a two-cell structure under oval shape with a circulation velocity ($\psi_{\max }$ = 12.3). For this case, isotherms appear as a thermal plume characterized by a distortion of the temperature profiles. When the flow is subjected to a constant magnetic field characterized by a Hartman number (Ha ≠ 0), both thermal and hydrodynamic fields in the enclosure show that the magnetic field causes a decrease in the flow force intensity. In other words, it appears that the magnetic field has the ability to slow down and attenuate convection currents. Knowing that as Ha increases, the streamlines become narrower and have a lower flow intensity, (e.g., for Ha = 25, $\psi_{\max }$ = 12, for Ha = 50, $\psi_{\max }$ = 11.2, and for Ha = 100, $\psi_{\max }$ = 9.4). This indicates that the magnetic field has a retarding effect on convection development. In its turn the isentropic profiles reveal that the entropy production in the enclosure declines with growing Hartman number, (e.g., for Ha = 25, S_max_ = 124.7 × 10^3^ and for Ha = 100, S_max_ = 83.3 × 10^3^). Therefore, we can say that the magnetic field is used to confine the convection, therefore the thermal losses in the concerned system become lower.

#### Darcy number effect

**Figure 5:**
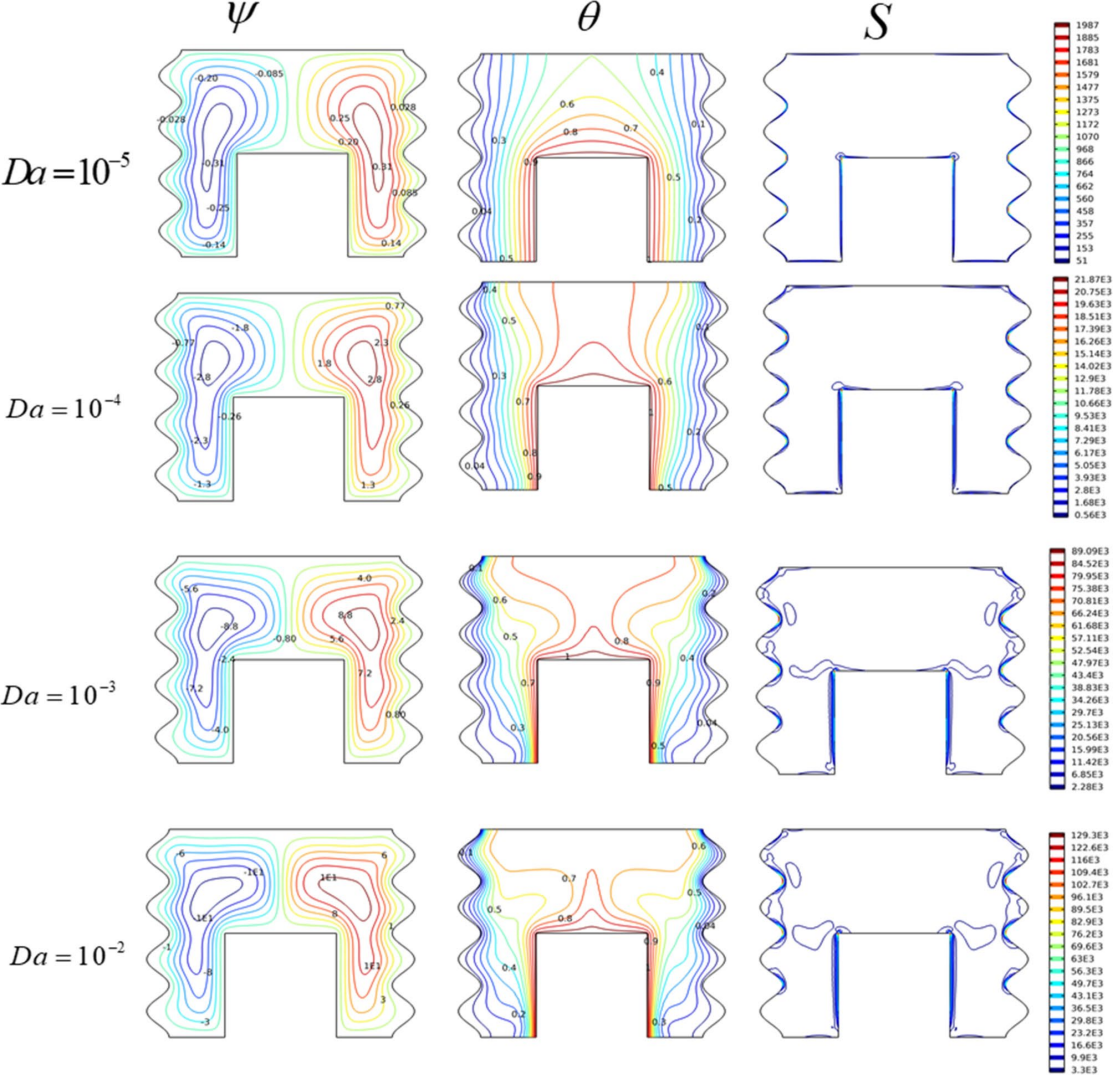
The impact of Da on (ψ) Streamlines, (θ) Isotherms, and (S) Entropy for Ha = 0, Ra = 10^6^.

Permeability of the porous media is considered using various values of Darcy number (10^–5^ $\le$ Da $\le$ 10^–2^) for (Ra = 10^6^; Ha = 0 and AR = 0.5). Figure 5 shows the Darcy number effects on current lines, temperature distribution, and isentropic lines in the odd-shaped cavity. High values of Da denote high permeability of the porous media, reflected by higher fluid velocities (e.g., for Da = 10^–2^, $\psi_{\max }$ = 12.32). In this case, the isotherms have a deformed shape with a thermal plume at the heated wall and a uniform temperature zone in the center of the cavity. Therefore, the entropy generation in this case is more important. As Da decreases, the ability of the porous medium to allow the fluid to pass through decreases, therefore the fluid slows down (for Da = 10^–5^, $\psi_{\max }$ = 0.32), furthermore, the convection rollers tend to tighten. The isotherms are stratified and reappear in uniform way within the cavity.

#### Heated-wall length effect

**Figure 6:**
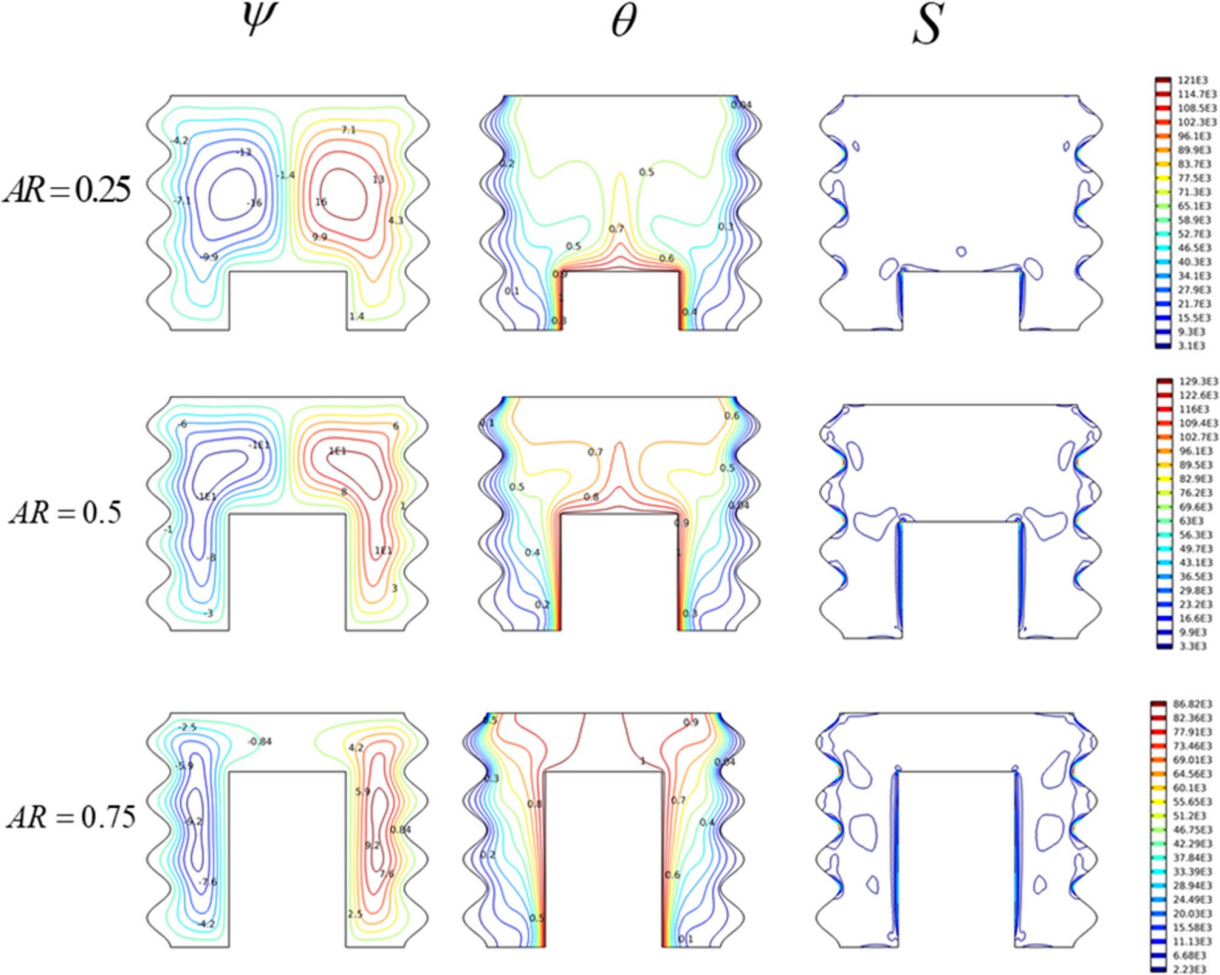
The impact of AR on (ψ) Streamlines, (θ) Isotherms, and (S) Entropy for Ha = 0, Ra = 10^6^.

In this part, effects of the geometrical shape of the cavity were presented using (0.5 $\le$ AR $\le$ 0.75) for (Ra = 10^6^, Ha = 0, and Da = 10^–2^). The results have been presented in Fig. 6, from this figure it was found that this parameter can play an important role on temperature repartition and streaming pattern as well as entropy production. Indeed, for AR = 0.25, the fluid flow area becomes larger, which induces bigger convection rollers and higher flow velocities ($\psi_{\max }$ = 16.2). As a result, the uniform temperature zone becomes larger, and a low entropy generation was observed. Concerning the situation of AR = 0.5, findings have been already commented on in the previous sections. The increasing value of AR = 0.75 narrows the surface area of the fluid. This induces convective cells with thin widths, consequently, the fluid velocity weakens ($\psi_{\max }$ = 9.2). As a result, the isotherms cluster on both sides of the cavity. In this case, the induced entropy has low values compared to the other two cases.

#### Heat transfer performance of odd-shaped enclosure

**Figure 7:**
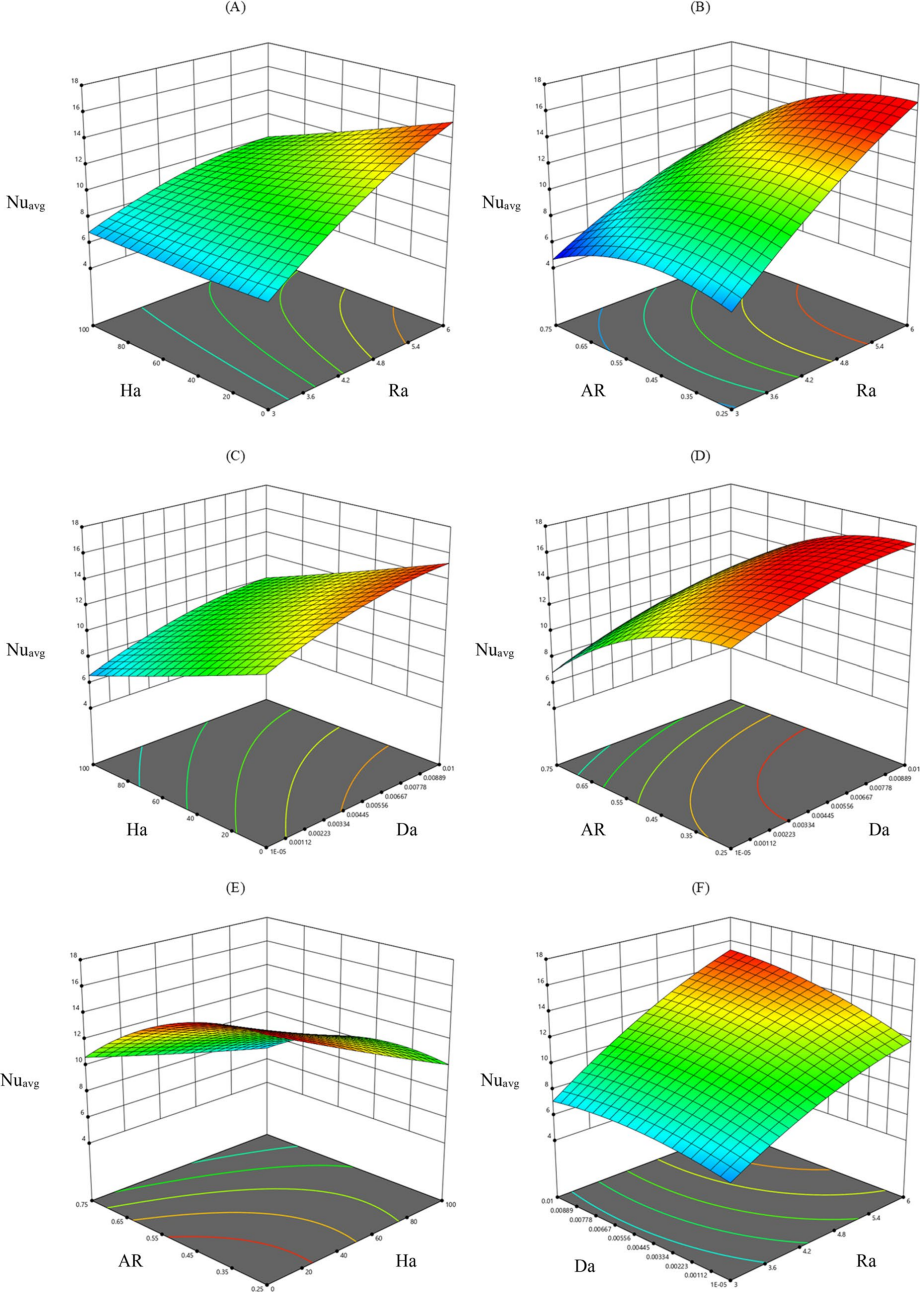
The variation of average Nu in various parameters.

For the characterization of heat transfer inside the porous cavity, Nu_avg_ has been reported as a function of previously defined control parameters. The results were given as 3-D curves, where the mean Nu is given versus two parameters in the same curve (see Fig. 7). A growing change of the Nusselt number has been recorded in accordance to the rising of Rayleigh number independently of the other parameters' values (see Fig. 7A,B,F). This is obvious given that the convection regime dominates heat transfer as Ra becomes higher. As introduced in the previous sections, increasing the number of Hartman's delays the fluid flow. Consequently, the rate of heat transfer in the cavity is translated by the decreasing evolution of the mean Nusselt number versus the Ha number (see Fig. 7A,C,E). From these three curves, it can be noted that the decrease of Nusselt number vs. Ha, is more remarkable for higher Rayleigh numbers, as well as higher Darcy numbers (see Fig. 7A,C). Also, it is interesting to note that Darcy's number has a positive effect on the change of Nu number. Indeed, the "increase in Da leads to an improvement of the heat transfer rate. Reducing the heating length has also a positive impact on mean Nusselt, or, as demonstrated in (Fig. 7B,D,E), when AR reduces the Nusselt number reaches higher values.

#### Characterization of entropy production

**Figure 8:**
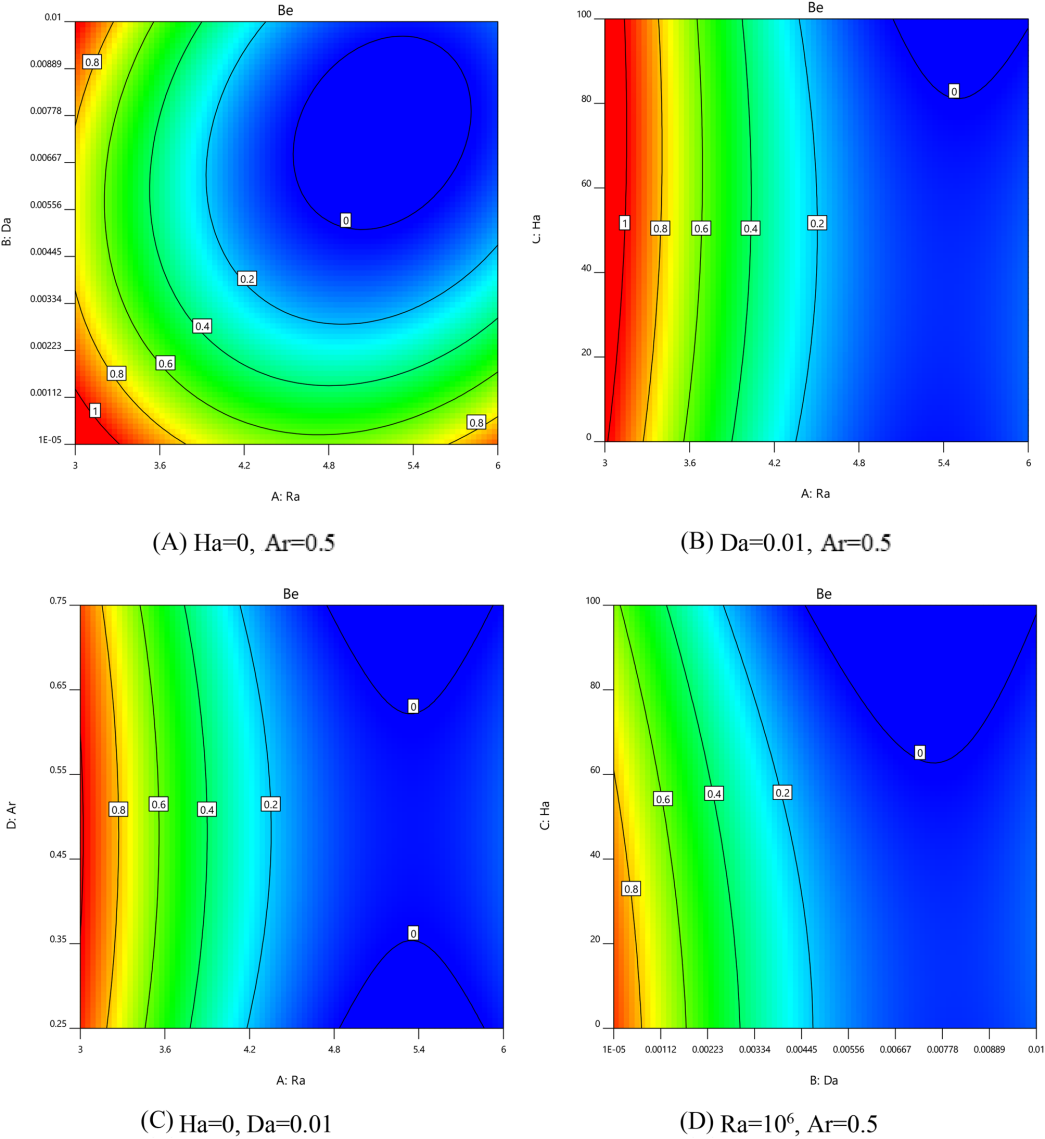
The variation Bejan number with various parameters.

To understand and characterize the energy losses within the cavity, Bejan number evolutions have been presented according to the control parameters. Combined effects of (Ra, Da); (Ra, Ha); (Ra, AR), and (Da, Ha) on Be number have be shown respectively in Fig. 8A–D. A cylindrical stratification variation was observed on the Bejan number behavior when Ra and Da are varied simultaneously (see Fig. 8A). From this figure, it can be seen that the Bejan number is highest at low Ra and regardless of Da. In this situation, entropy occurs mainly by thermal irreversibility. This scenario has also been observed when Ra reaches its maximum with a combination with lower Da. For higher values of Ra and Da together, irreversibility’s due to the friction forces are predominant. Concerning the effects of combination (Ra, Ha) on Be, it turned out that Be's number moved per vertical segment according to Ha as a function of Rayleigh number (see Fig. 8B). As Ra becomes higher, Be tends to become lower, whatever the magnetic field intensity is considered (Ha). In other words, an almost constant evolution of Be as a function of the Ha number. But Ha's effects on Be are more remarkable for small values of Da (see Fig. 8D), meaning that as Ha increases, energy degradation in a cavity is mainly due to temperature gradient effects. On another side, the effects of Ar parameter combined with the Rayleigh number (Fig. 8C), demonstrate that the geometric parameter Ar has little effect on Bejan number variations.

## Conclusion

We have presented empirical parametric research on natural convection and entropy formation inside a cavity filled with a nanofluid exposed to a magnetic field under the presence of a porous medium. It is possible to summarize the most relevant points arising from this work as follows:

- The heat transfer in the cavity is affected by flow regime (Ra), magnetic field strength Ha; medium porosity Da and heating length characterized by Ar. Note that the effects level of each parameter mentioned differs from the other.
- Reducing the Da number leads to a drop in flow intensity.
- The thermal exchange rate within the oddly shaped cavity improves with increasing Ra.
- The reduction in Nusselt numbers relative to Ha is more noticeable for higher Rayleigh numbers, as well as for higher Darcy numbers.
- Entropy generation within the cavity increases with increased Ra and diminishes at higher Ha values.
- Hartman number effects on Bejan number are most noticeable for small quantities of Darcy's number.

## Data Availability

The results of this study are available only within the paper to support the data.

## Abbreviations

h: Dimensional length of the heated wall (m)
H: Height of the outer and inner conical (m)
Nu: Nusselt number
p: Static pressure (N/m^2^)
P: Dimensionless pressure
Pr: Prandtl number
Ra: Rayleigh number
X,Y: Dimensionless coordinate
T: Local temperature (^°^K)
u: Velocity in the x direction (m/s)
v: Velocity in the y direction (m/s)
U,V: Dimensionless velocity

## Greek symbols

α: Thermal diffusivity (m^2^/s)
*Β*: Coefficient of thermal expansion (1/^°^K)
$\varepsilon$: Dimensionless size of the heater
$$\theta$$: Dimensionless temperature
ρ: Fluid density (kg /m^3^)
ν: Kinematic viscosity (m^2^ /s)
μ: Dynamic viscosity (kg/ms)
λ: Fluid thermal conductivity (w /m ^°^K)

## References

[1] Choi SUS (1995). Enhancing thermal conductivity of fluids with nanoparticles. Am. Soc. Mech. Eng. Fluids Eng. Div..

[2] Meena P, Tammasaeng P, Kanphirom J, Ponkho A, Setwong S (2014). Enhancement of the performance heat transfer of a thermosyphon with fin and without fin heat exchangers using Cu-nanofluid as working fluids. J. Eng. Thermophys..

[3] Hasona WM (2019). Temperature-dependent viscosity and thermal conductivity effects on peristaltic flow of Carreau-Yasuda nanofluid in a 2D tapered asymmetric channel: Applications of solar collectors. Mech. Time-Dependent Mater..

[4] Hanif H, Khan I, Shafie S (2020). A novel study on time-dependent viscosity model of magneto-hybrid nanofluid flow over a permeable cone: Applications in material engineering. Eur. Phys. J. Plus.

[5] Li SF, Wang PY, Hua Liu Z (2018). A basic study on Thermosyphon-type thermal storage unit (TSU) using Nanofluid as the heat transfer medium. Heat Mass Transf. Stoffuebertragung.

[6] Ali F, Murtaza S, Khan I, Sheikh NA, Nisar KS (2019). Atangana–Baleanu fractional model for the flow of Jeffrey nanofluid with diffusion-thermo effects: applications in engine oil. Adv. Differ. Equations.

[7] Khan SU, Ali HM (2020). Swimming of gyrotactic microorganisms in unsteady flow of eyring powell nanofluid with variable thermal features: some bio-technology applications. Int. J. Thermophys..

[8] Khan I, Hussanan A, Saqib M, Shafie S (2019). Convective heat transfer in drilling nanofluid with clay nanoparticles: Applications in water cleaning process. Bionanoscience.

[9] Sahoo RR (2020). Thermo-hydraulic characteristics of radiator with various shape nanoparticle-based ternary hybrid nanofluid. Powder Technol..

[10] Choi TJ, Kim SH, Jang SP, Yang DJ, Byeon YM (2020). Heat transfer enhancement of a radiator with mass-producing nanofluids (EG/water-based Al2O3 nanofluids) for cooling a 100 kW high power system. Appl. Therm. Eng..

[11] Ahmadi MR, Toghraie D (2021). Numerical analysis of flow and heat transfer in a shell and tube heat exchanger in the gas recirculation cooling system of a diesel engine and the effect of nanofluid on its performance. J. Therm. Anal. Calorim..

[12] El Desouky AA, Ismail HNA, Abourabia AM, Ahmed NA (2020). Numerical simulation of MHD flow and heat transfer inside T-shaped cavity by the parallel walls motion. SN Appl. Sci..

[13] Martínez-Merino P, Sánchez-Coronilla A, Alcántara R, Martín EI, Navas J (2020). Insights into the stability and thermal properties of WSe2-based nanofluids for concentrating solar power prepared by liquid phase exfoliation. J. Mol. Liq..

[14] Fadodun OG, Amosun AA, Olaloye DO (2021). Numerical modeling of entropy production in Al2O3/H2O nanofluid flowing through a novel Bessel-like converging pipe. Int. Nano Lett..

[15] Mourad A, Aissa A, Mebarek-Oudina F, Al-Kouz W, Sahnoun M (2021). Natural convection of nanoliquid from elliptic cylinder in wavy enclosure under the effect of uniform magnetic field: Numerical investigation. Eur. Phys. J. Plus.

[16] Aissa A, Amine Medebber M, Al-Farhany K, Sahnoun M, Khaleel Kareem A, El Ganaoui M (2020). Effect of magnetic field on nanofluid free convection in conical partially annular space. MATEC Web Conf.

[17] Al-Kouz W, Al-Muhtady A, Owhaib W, Al-Dahidi S, Hader M, Abu-Alghanam R (2019). Entropy generation optimization for rarified nanofluid flows in a square cavity with two fins at the hot wall. Entropy.

[18] Abdelrazik AS, Tan KH, Aslfattahi N, Arifutzzaman A, Saidur R, Al-Sulaiman FA (2020). Optical, stability and energy performance of water-based MXene nanofluids in hybrid PV/thermal solar systems. Sol. Energy.

[19] Beriache M, Sidik NAC, Yazid MNAWM, Mamat R, Najafi G, Kefayati GHR (2016). A review on why researchers apply external magnetic field on nanofluids. Int. Commun. Heat Mass Transf..

[20] H. Darcy, Les fontaines publiques de la ville de Dijon: Exposition et application des principes a suivre et des formules a employer dans les questions de distribution d’eau; ouvrage terminé par un appendice relatif aux fournitures d’eau de plusieurs villes au filtr. Victor Dalmont, Libraire des Corps imperiaux des ponts et chaussées et des mines, 1856.

[21] Shahsavar A, Entezari S, Toghraie D, Barnoon P (2020). Effects of the porous medium and water-silver biological nanofluid on the performance of a newly designed heat sink by using first and second laws of thermodynamics. Chin. J. Chem. Eng..

[22] Alihosseini S, Jafari A (2020). The effect of porous medium configuration on nanofluid heat transfer. Appl. Nanosci..

[23] Benos LT, Polychronopoulos ND, Mahabaleshwar US, Lorenzini G, Sarris IE (2021). Thermal and flow investigation of MHD natural convection in a nanofluid-saturated porous enclosure: An asymptotic analysis. J. Therm. Anal. Calorim..

[24] Baïri A, Alilat N (2020). Thermal design of a spherical electronic device naturally cooled by means of water–copper nanofluid saturated porous media. J. Therm. Anal. Calorim..

[25] Liu X, Toghraie D, Hekmatifar M, Akbari OA, Karimipour A, Afrand M (2020). Numerical investigation of nanofluid laminar forced convection heat transfer between two horizontal concentric cylinders in the presence of porous medium. J. Therm. Anal. Calorim..

[26] Tahmasbi M, Siavashi M, Abbasi HR, Akhlaghi M (2020). Mixed convection enhancement by using optimized porous media and nanofluid in a cavity with two rotating cylinders. J. Therm. Anal. Calorim..

[27] Shafee A, Rezaeianjouybari B, Tlili I (2021). Treatment of nanofluid within porous media using non-equilibrium approach. J. Therm. Anal. Calorim..

[28] Salari M, Assari MR, Ghafouri A, Pourmahmoud N (2020). Experimental study on forced convection heat transfer of a nanofluid in a heat exchanger filled partially porous material. J. Therm. Anal. Calorim..

[29] Aminian E, Moghadasi H, Saffari H (2020). Magnetic field effects on forced convection flow of a hybrid nanofluid in a cylinder filled with porous media: a numerical study. J. Therm. Anal. Calorim..

[30] Kefayati GHR, Tang H (2017). Simulation of natural convection and entropy generation of MHD non-Newtonian nanofluid in a cavity using Buongiorno’s mathematical model. Int. J. Hydrogen Energy.

[31] Afsana S, Molla MM, Nag P, Saha LK, Siddiqa S (2021). MHD natural convection and entropy generation of non-Newtonian ferrofluid in a wavy enclosure. Int. J. Mech. Sci..

[32] Bahiraei M, Mazaheri N, Daneshyar MR (2020). CFD analysis of second law characteristics for flow of a hybrid biological nanofluid under rotary motion of a twisted tape: Exergy destruction and entropy generation analyses. Powder Technol..

[33] Bahiraei M, Jamshidmofid M, Dahari M (2020). Second law analysis of hybrid nanofluid flow in a microchannel heat sink integrated with ribs and secondary channels for utilization in miniature thermal devices. Chem. Eng. Process. Process Intensif..

[34] Ma Y, Shahsavar A, Talebizadehsardari P (2020). Two-phase mixture simulation of the effect of fin arrangement on first and second law performance of a bifurcation microchannels heatsink operated with biologically prepared water-Ag nanofluid. Int. Commun. Heat Mass Transf..

[35] Xu YJ, Bilal M, Al-Mdallal Q, Khan MA, Muhammad T (2021). Gyrotactic micro-organism flow of Maxwell nanofluid between two parallel plates. Sci. Rep..

[36] Muhammad T, Waqas H, Khan SA, Ellahi R, Sait SM (2021). Significance of nonlinear thermal radiation in 3D Eyring-Powell nanofluid flow with Arrhenius activation energy. J. Therm. Anal. Calorim..

[37] Muhammad T, Alamri SZ, Waqas H, Habib D, Ellahi R (2021). Bioconvection flow of magnetized Carreau nanofluid under the influence of slip over a wedge with motile microorganisms. J. Therm. Anal. Calorim..

[38] Muhammad T, Waqas H, Farooq U, Alqarni MS (2021). Numerical simulation for melting heat transport in nanofluids due to quadratic stretching plate with nonlinear thermal radiation. Case Stud. Thermal Eng..

[39] Tayebi T, Chamkha AJ (2021). Effects of various configurations of an inserted corrugated conductive cylinder on MHD natural convection in a hybrid nanofluid-filled square domain. J. Therm. Anal. Calorim..

[40] Dogonchi AS, Tayebi T, Karimi N, Chamkha AJ, Alhumade H (2021). Thermal-natural convection and entropy production behavior of hybrid nanoliquid flow under the effects of magnetic field through a porous wavy cavity embodies three circular cylinders. J. Taiwan Inst. Chem. Eng..

[41] Tayebi T, Chamkha AJ, Melaibari AA, Raouache E (2021). Effect of internal heat generation or absorption on conjugate thermal-free convection of a suspension of hybrid nanofluid in a partitioned circular annulus. Int. Commun. Heat Mass Transfer.

[42] Tayebi T, Dogonchi AS, Karimi N, Ge-JiLe H, Chamkha AJ, Elmasry Y (2021). Thermo-economic and entropy generation analyses of magnetic natural convective flow in a nanofluid-filled annular enclosure fitted with fins. Sustain. Energy Technol. Assess..

[43] Xiong Q, Altnji S, Tayebi T, Izadi M, Hajjar A, Sundén B, Li LK (2021). A comprehensive review on the application of hybrid nanofluids in solar energy collectors. Sustain. Energy Technol. Assess..

[44] Hussien AA, Al-Kouz W, Yusop NM, Abdullah MZ, Janvekar AA (2019). A brief survey of preparation and heat transfer enhancement of hybrid nanofluids. Strojniski Vestnik J. Mech. Eng..

[45] Al-Kouz W, Bendrer BAI, Aissa A, Almuhtady A, Jamshed W, Nisar KS, Zakarya M (2021). Galerkin finite element analysis of magneto two-phase nanofluid flowing in double wavy enclosure comprehending an adiabatic rotating cylinder. Sci. Rep..

[46] Abu-Libdeh N, Redouane F, Aissa A, Mebarek-Oudina F, Almuhtady A, Jamshed W, Al-Kouz W (2021). Hydrothermal and entropy investigation of Ag/MgO/H2O hybrid nanofluid natural convection in a novel shape of porous cavity. Appl. Sci..

[47] Mahanthesh B, Mackolil J, Radhika M, Al-Kouz W (2021). Significance of quadratic thermal radiation and quadratic convection on boundary layer two-phase flow of a dusty nanoliquid past a vertical plate. Int. Commun. Heat Mass Transfer.

[48] Al-Kouz W, Al-Waked R, Sari ME, Owhaib W, Atieh A (2018). Numerical study of heat transfer enhancement in the entrance region for low-pressure gaseous laminar pipe flows using Al2O3–air nanofluid. Adv. Mech. Eng..

[49] Mukhtar T, Jamshed W, Aziz A, Al-Kouz W (2020). Computational investigation of heat transfer in a flow subjected to magnetohydrodynamic of Maxwell nanofluid over a stretched flat sheet with thermal radiation. Numer. Methods Partial Differ. Equ..

[50] Izadi M, Mohebbi R, Karimi D, Sheremet MA (2018). Numerical simulation of natural convection heat transfer inside a shaped cavity filled by a MWCNT-Fe3O4/water hybrid nanofluids using LBM. Chem. Eng. Process. Process Intens..

[51] Abdel-Nour Z, Aissa A, Mebarek-Oudina F, Rashad AM, Ali HM, Sahnoun M, El Ganaoui M (2020). Magnetohydrodynamic natural convection of hybrid nanofluid in a porous enclosure: Numerical analysis of the entropy generation. J. Therm. Anal. Calorim..

[52] Zaim A, Aissa A, Mebarek-Oudina F, Mahanthesh B, Lorenzini G, Sahnoun M, El Ganaoui M (2020). Galerkin finite element analysis of magneto-hydrodynamic natural convection of Cu-water nanoliquid in a baffled U-shaped enclosure. Propuls. Power Res..

[53] Jamshed W (2021). Numerical investigation of MHD impact on maxwell nanofluid. Int. Commun. Heat Mass Transf..

[54] Jamshed W, Nisar KS (2021). Computational single phase comparative study of williamson nanofluid in parabolic trough solar collector via keller box method. Int. J. Energy Res.

[55] Jamshed W, Eid MR, Mohd Nasir NAA, Nisar KS, Aziz A, Shahzad F, Saleel CA, Shukla A (2021). Thermal examination of renewable solar energy in parabolic trough solar collector utilizing Maxwell nanofluid: A noble case study. Case Stud. Therm. Eng..

[56] Jamshed W, Mishra SR, Pattnaik PK, Nisar KS, Devi SSU, Prakash M, Shahzad F, Hussain M, Vijayakumar V (2021). Features of entropy optimization on viscous second grade nanofluid streamed with thermal radiation: A Tiwari and Das model. Case Stud. Therm. Eng..

[57] Jamshed W, Akgül EK, Nisar KS (2021). Keller box study for inclined magnetically driven Casson nanofluid over a stretching sheet: single phase model. Phys. Scr..

[58] Calcagni B, Marsili F, Paroncini M (2005). Natural convective heat transfer in square enclosures heated frombelow. Appl. Therm. Eng..

